# The role of ROS and subsequent DNA-damage response in PUMA-induced apoptosis of ovarian cancer cells

**DOI:** 10.18632/oncotarget.15626

**Published:** 2017-02-22

**Authors:** Jun Yang, Xinyu Zhao, Mei Tang, Lei Li, Yi Lei, Ping Cheng, Wenhao Guo, Yu Zheng, Wei Wang, Na Luo, Yong Peng, Aiping Tong, Yuquan Wei, Chunlai Nie, Zhu Yuan

**Affiliations:** ^1^ State Key Laboratory of Biotherapy and Cancer Center, West China Hospital, Sichuan University/Collaborative Innovation Center of Biotherapy, West China Hospital, Sichuan University, Chengdu 610041, China; ^2^ Department of Abdominal Oncology, Cancer Center and State Key Laboratory of Biotherapy, West China Hospital, West China Medical School, Sichuan University, Chengdu 610041, Sichuan Province, China; ^3^ Nankai University, School of Medicine/Collaborative Innovation Center of Biotherapy, Tianjin 300071, China

**Keywords:** PUMA, ROS, apoptosis, DNA damage response, ovarian cancer

## Abstract

PUMA is a member of the “BH3-only” branch of the BCL-2 family. Our previous study suggests a therapeutic potential of PUMA in treating ovarian cancer, however, the action mechanism of PUMA remains elusive. In this work, we found that in PUMA adenovirus-infected A2780s ovarian cancer cells, exogenous PUMA was partially accumulated in the cytosol and mainly located to the mitochondria. We further showed that PUMA induces mitochondrial dysfunction-mediated apoptosis and ROS generation through functional BAX in a ROS generating enzyme- and caspase-independent manner irrespective of their p53 status, and results in activation of Nrf2/HO-1 pathway. Furthermore, PUMA induces DNA breaks in γ-H2AX staining, and causes activation of DNA damage-related kinases including ATM, ATR, DNA-PKcs, Chk1 and Chk2, which are correlated with the apoptosis. PUMA also results in ROS-triggered JNK activation. Intriguingly, JNK plays a dual role in both DNA damage response and apoptosis, and has an additional contribution to apoptosis. Taken together, we have provided new insight into the action mechanism by which elevated PUMA first induces ROS generation then results in DNA damage response and JNK activation, ultimately contributing to apoptosis in ovarian cancer cells.

## INTRODUCTION

Ovarian cancer is one type of lethal gynecologic cancer [1, 2]. Because ovarian cancer often has no symptom at the early stage, most of patients are diagnosed at the late stages [3]. Although treatment with platinum and paclitaxel can prolong survival to some extent, drug resistance often leads to therapy failure [4, 5]. Nevertheless, some novel molecular targets have been discovered for treating ovarian cancer [6–9].

Recent reports suggest that deregulations in apoptotic pathways are associated with cancer progress [9, 10]. P53 mutation often causes resistance to chemotherapy [11, 12]. Previous studies showed that p73 is an important determinant for chemosensitivity [12–14]. These observations showed that alteration of p53- or p73-related apoptotic signaling promotes cancer progress and affects chemotherapeutic efficacy [12]. Therefore, restoration of the deregulated p53 or p73 pathway might be an effective method to enhance the chemotherapeutic efficacy [12].

PUMA was a transcriptional target of p53 as well as p73, and could induce apoptosis of many kinds of cancer cells [2, 12, 15, 16]. PUMA, one member of the Bcl-2 family, has one “BH3-only” domain [2, 10, 17]. PUMA is localized in the mitochondria and triggers mitochondrial dysfunction mediated-apoptosis [2, 18, 19]. It antagonizes the functions of BCL-XL and MCL-1 through Bax/Bak [2, 18, 19]. Previous studies suggested that PUMA could inhibit tumor growth [2, 20, 21]. Furthermore, PUMA alone or combined with irradiation or other chemotherapy drugs, showed strong antitumor efficacy on many types of cancers [2, 22–25]. Our previous studies suggested a potential application of PUMA for treating ovarian cancer [2, 12]. However, the action mechanism remains unclear.

ROS was generated when PUMA was overexpressed in colorectal tumor cells [26]. However, the role of ROS remains unclear. The purpose of this work is to investigate if PUMA also results in ROS generation, and further elucidate the role of ROS and subsequent DNA-damage response in PUMA-induced apoptosis.

In this work, considering that PUMA is a transcript target of p53/p73, we first selected A2780s, OVCAR-3, SKOV3 and A2780cp ovarian cancer cells as human cell models because these cells have different p53 status, that is, their p53 status are p53 wild-type, p53 mutant, p53−/− and p53 wild-type (loss of p53 function), respectively [2, 12, 27, 28]. Then we constructed a recombinant adenovirus expressing PUMA (Ad-PUMA) to investigate the suppressive effects of Ad-PUMA on cell growth, as well as elucidated the role of ROS in PUMA-induced apoptosis. Similar to the previous report [26], in Ad-PUMA-infected ovarian cancer cells, we also observed ROS generation. We further found that ROS induced subsequent DNA damage response and JNK activation, which contributed to the apoptosis. To our knowledge, we report a new proapoptotic mechanism for PUMA.

## RESULTS

### PUMA inhibits cell proliferation

**Figure 1:**
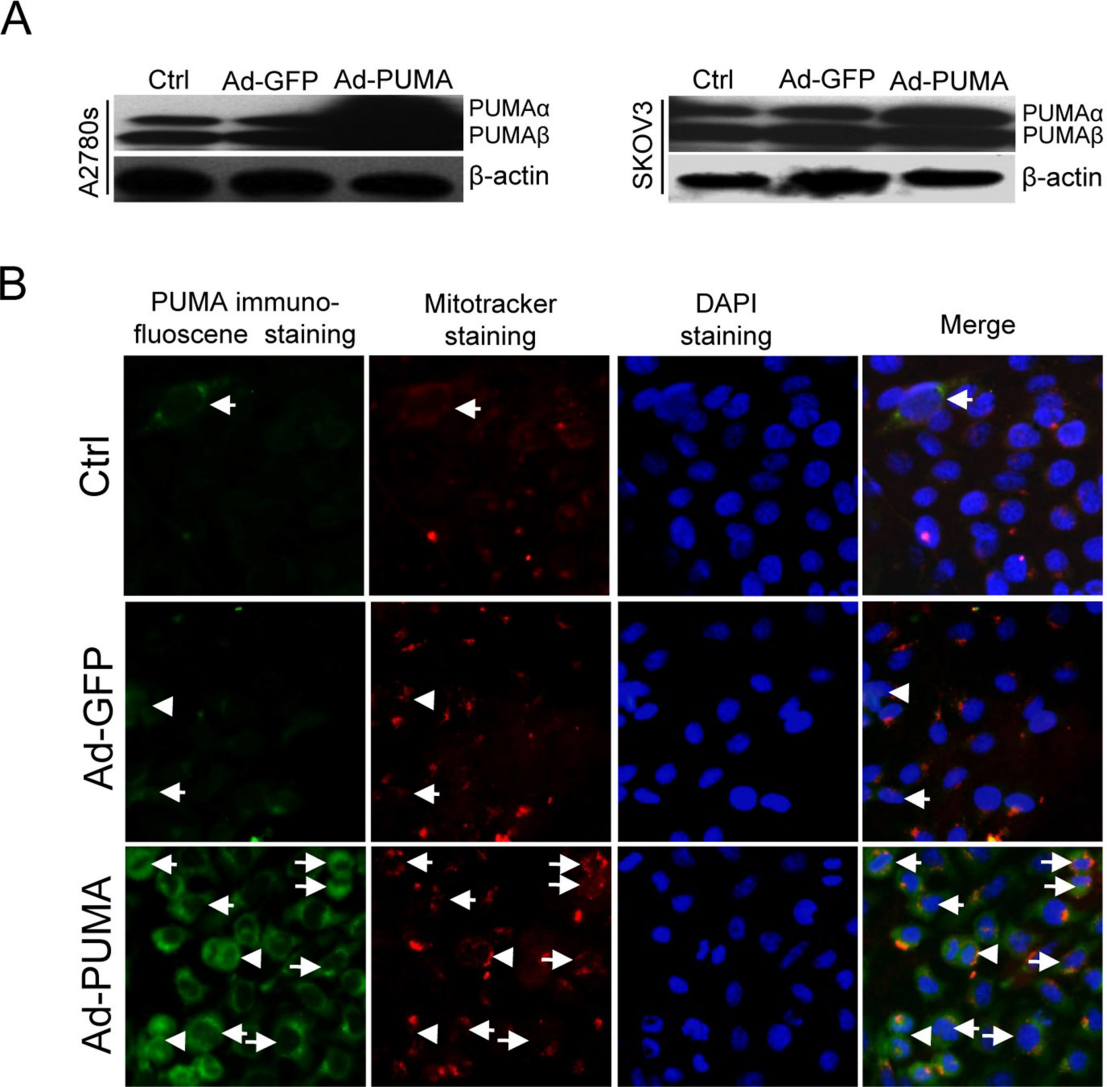
Subcellular localization of exogenous PUMA (**A**) Western blotting analysis of PUMA overexpression in A2780s and SKOV3 cells infected with PUMA adenovirus for 36 h. β-actin was used as a loading control. (**B**) SKOV3 cells were infected with Ad-PUMA adenovirus for 36 h, and then the subcellular localization of PUMA was analyzed by merging the images of immunofluorescence staining with PUMA antibodies and that of mitotracker staining. Exogenous PUMA was partially accumulated in the cytosol and mainly located in the mitochondria. Arrows represent mitochondrial localization of PUMA whereas arrowheads represent typical cytosol localization.

Our previous work showed that elevated expression of PUMA using plasmid transfection method led to growth suppression of ovarian cancer cells [2]. In this work, we have constructed the PUMA adenoviruses (Ad-PUMA), which caused the morphology change of 293A cells (Supplementary Figure 1A and 1B). Considering that PUMA is a p53 downstream target, we selected several ovarian cancer cell lines as cell models to investigate the effects of Ad-PUMA on ovarian cancer cell proliferation according to our previous report [12]. These cells were infected with adenovirus PUMA (Ad-PUMA) or control adenovirus (Ad-GFP). For all four cell lines, at least 80% of the cells were infected by adenovirus as indicated by the GFP signal (data not shown). Overexpression of PUMA was further confirmed by western blot (Figure 1A). Notably, because two transcription variants of PUMA including PUMAα and PUMA*β* exist, we detected two bands by western blot using anti-PUMA antibody. In this work, we used PUMAα to construct the recombinant adenovirus and named it as Ad-PUMA.

A recent report has shown that due to its localization in the cytosol, neither upregulation nor overexpression of PUMA was associated with cell death, whereas some pro-apoptotic factors can promote PUMA to translocate into the mitochondria, resulting in apoptosis [29]. These observations suggested that accumulation in the cytosol and translocation to the mitochondria might be vital for the function of PUMA. As expected, in SKOV3 cells infected with Ad-PUMA or Ad-GFP adenovirus for 48 h, the expression of exogenous PUMA was elevated significantly than that of control and GFP adenovirus group cells (Figure 1A). Furthermore, exogenous PUMA was partially accumulated in the cytosol and mainly located to the mitochondria (Figure 1B).

Furthermore, PUMA significantly reduced the viability of A2780s, SKOV3, OVCAR3 and A2780cp cells as evidenced by MTT assay (Supplementary Figure 1C) and colony formation assays (Supplementary Figure 1D).

### PUMA induces apoptosis via mitochondrial apoptotic pathway

Considering that the action of PUMA might be affected by p53 status, we mainly selected A2780s and SKOV3 cells in the following experiments to elucidate the underlying action mechanism of PUMA. Several lines of evidences have shown that apoptosis is vital for reducing cell viability by PUMA [2, 15, 19, 22–24]. Similarly, exogenous PUMA induced significant apoptosis of A2780s and SKOV3 cells infected with Ad-PUMA for 60 h, as evidenced by the flow cytometry analysis and detection of caspase-3 activity (Supplementary Figure 2A–2D). Furthermore, the apoptosis results from decrease of the mitochondrial membrane potential (Supplementary Figure 2E and 2F).

### PUMA induces mitochondria ROS generation through functional BAX

**Figure 2:**
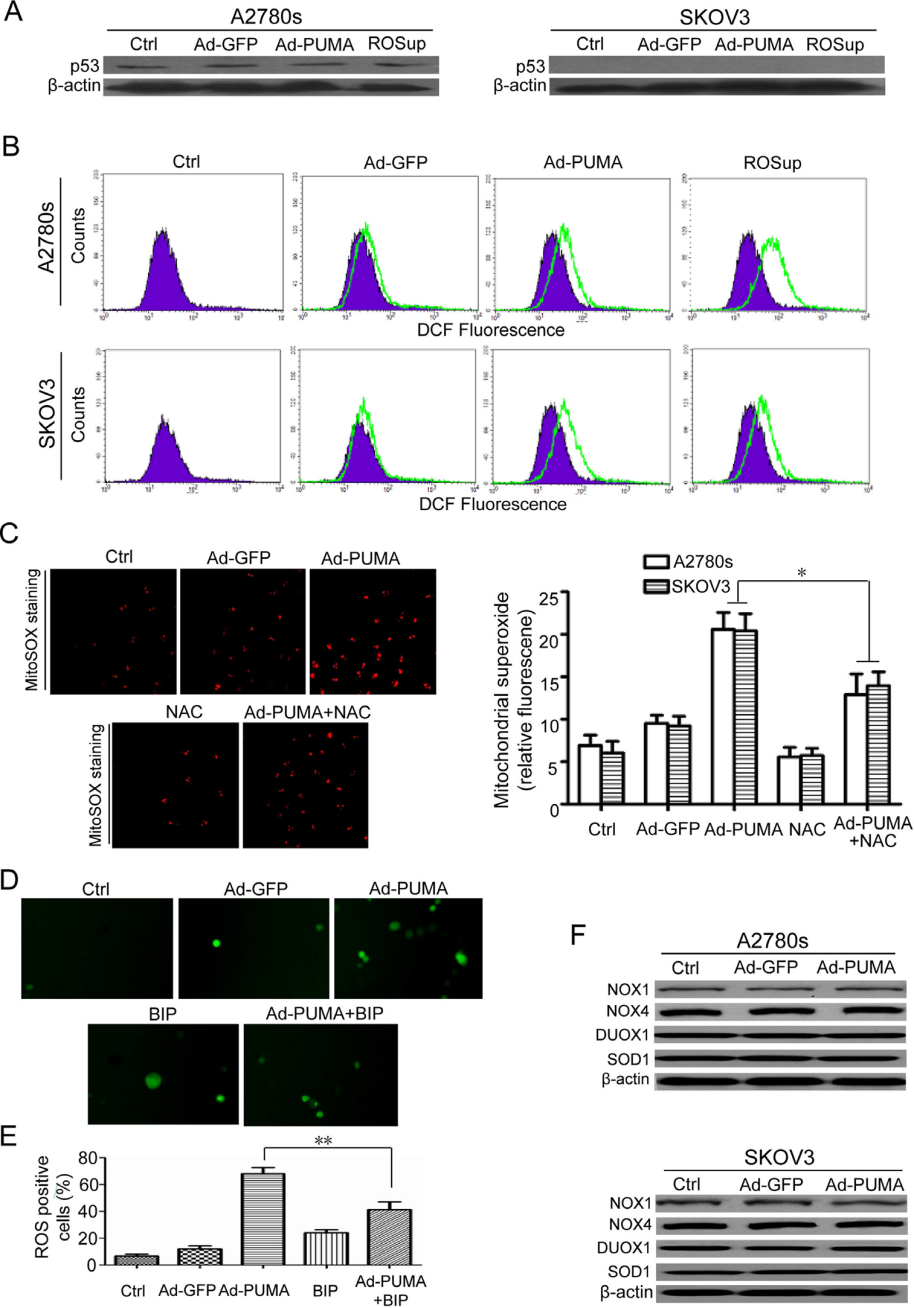
PUMA induces mitochondria ROS generation through functional BAX (**A**) p53 wild-type A2780s and p53-null SKOV3 cells were untreated or infected with Ad-GFP or Ad-PUMA for 36 h, and then the expressions of p53 were detected by western blotting. β-actin was used as a loading control. (**B**) Measurement of ROS. A2780s and SKOV3 cells were untreated or treated with ROSup (to provide a positive control) or infected with Ad-GFP or Ad-PUMA for 36h. The treated cells were then used for measuring ROS level by DCF fluorescence with flow cytometry. (**C**) A2780s and SKOV3cells were treated as described in B, and then mitochondrial ROS generation was determined by a MitoSOX red mitochondrial superoxide indicator. Representative MitoSOX red mitochondrial fluorescence staining pictures of SKOV3 cells were shown (left panel). NAC significantly abrogated the MitoSOX fluorescence intensity of A2780s and SKOV3 cells induced by PUMA (right panel). Bars, mean; error bars, S.D. (*n* = 3, *p < 0.05). (**D**) Blocking of ROS by a BAX-inhibiting peptide (BIP). SKOV3 cells were infected with Ad-PUMA for 24h, then treated with BIP for another 12 h. The treated cells were then used for measuring ROS generation by DCFH fluorescence staining. The representative pictures of PUMA and/or BIP-induced ROS generation in SKOV3 cells were shown. (**E**) Quantification of ROS positive cells in D. The ROS positive cells (at least 100 total cells) were counted. Bars, mean; error bars, S.D. (*n* = 3; **p < 0.01). (**F**) A2780s and SKOV3 cells were untreated or infected with Ad-PUMA for 36 h, and then the expressions of NOX1, NOX4, DUOX1 and SOD1 were detected by western blotting. β-actin was used as a loading control.

2′7′-dichlorofluorescein diacetate was used to detect intracellular ROS change in A2780s and SKOV3 cells after infection with Ad-PUMA for 36h. We observed that the ROS generation had a significant increase both in A2780s (p53 wild-type) and SKOV3 (p53-null) cells (Figure 2A), as evidenced by flow cytometry analysis (Figure 2B), indicating that induction of ROS by PUMA does not require p53 expression.

ROS mainly originated in mitochondria and is a factor determining cell fate [30, 31]. The results of MitoSOX fluorescence staining showed that ROS generation mainly originated in mitochondria in A2780s cells after infection with PUMA adenovirus for 36 h, as indicated by N-Acetyl-L-cysteine (NAC) abrogated mitochondrial superoxide generation (Figure 2C). PUMA triggers mitochondrial dysfunction through BAX /BAK and promotes apoptosis by antagonizing BCL-XL and MCL-1 [2]. Furthermore, PUMA overexpression also induces BAX oligomerization in mitochondria [18, 19]. According to the previous reports [26, 32], we synthesized a BAX-inhibiting peptide (BIP) to investigate whether BAX was vital for PUMA-induced ROS generation. In this work, BIP markedly decreased ROS levels in A2780s and SKOV3 cells, as indicated by the fluorescence microscope (Figure 2D and 2E).

We further measured the expression levels of several ROS-related enzymes including NADPH oxidase 1 (NOX1), NOX4, DUOX1 and superoxide dismutase 1 (SOD1). As shown in Figure 2F, all these proteins remained unchanged in PUMA adenovirus-infected A2780s and SKOV3 cells, thus rule out the possibility that PUMA induces ROS generation in a ROS generating enzyme-dependent manner. Taken together, our findings showed that BAX is very important for ROS generation.

### PUMA induced ROS generation in a caspase-independent manner and activates the Nrf2/HO-1 pathway

**Figure 3:**
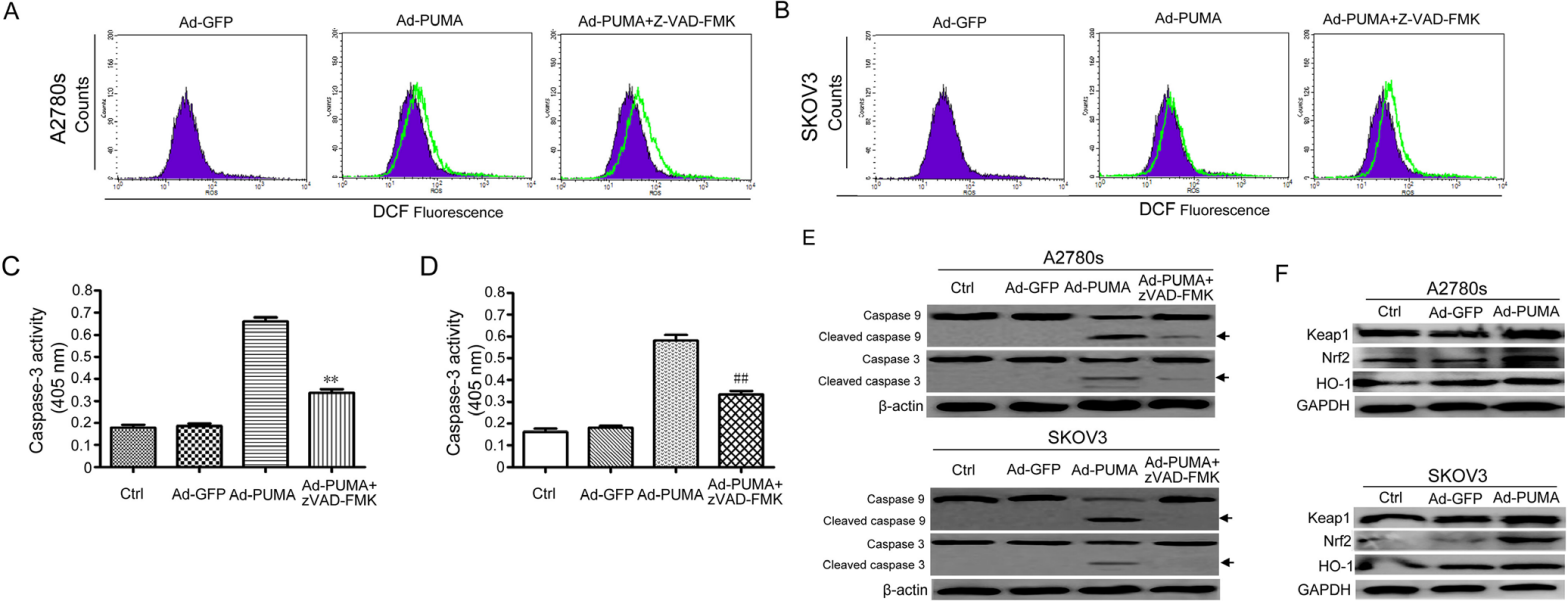
ROS is generated in a caspase-independent manner and activates the Nrf2/HO-1 pathway (**A**) A2780s cells were infected with Ad-PUMA for 36 h in the presence or absence of 10 μM benzyloxycarbonyl-VAD-fluoromethyl ketone (z-VAD-FMK). Then the treated cells were used for measuring ROS level by DCF fluorescence with flow cytometry. (**B**) SKOV3 cells were infected with Ad-PUMA for 36 h in the presence or absence of 10 μM benzyloxycarbonyl-VAD-fluoromethyl ketone (z-VAD-FMK). Then the treated cells were used for measuring ROS level by DCF fluorescence with flow cytometry. (**C**) Effect of zVAD-FMK on caspase-3 activity. A2780s cells were infected with Ad-GFP or Ad-PUMA for 48 h. The treated cells were then lysed and caspase 3 activity was measured using an assay kit (*n* = 3; **p < 0.01). (**D**) Effect of zVAD-FMK on caspase-3 activity. SKOV3 cells were infected with Ad-GFP or Ad-PUMA for 48 h. The treated cells were then lysed and caspase 3 activity was measured using an assay kit (*n* = 3; ^##^*p* < 0.01). (**E**) A2780s and SKOV3 cells were treated as described in A, and then cleavage of caspase-3 and -9 were detected by western blotting. β-actin was used as a loading control. (**F**) A2780s and SKOV3 cells were infected with Ad-PUMA for 36 h, and then the expressions of Nrf2, HO-1 and Keap1 were detected by western blotting. GAPDH was used as a loading control.

Both apoptosis and caspase activation can generate ROS [33]. Therefore, it was essential to investigate whether ROS increase is a cause or a consequence of caspase activation. Pretreatment of A2780s and SKOV3 cells with Z-VAD-FMK did not abrogate PUMA-induced ROS increase (Figure 3A and 3B), indicating that the observed increase in ROS is not a consequence of caspase activation. However, Z-VAD-FMK significantly decreased PUMA-induced caspase-3 activation (Figure 3C and 3D) and efficiently inhibits the cleavage of caspase-3 and -9 (Figure 3E).

The Nrf2/HO-1 axis regulates some key proteins which are responsible for protecting cells against oxidative stress [34]. Considering that PUMA-induced ROS generation might be attributable to inhibition of antioxidative signal, we further measured the levels of proteins involved in ROS production in A2780s and SKOV3 cells after infection with adenovirus for 48 h. Surprisingly, we observed that PUMA apparently up-regulated the levels of the transcription factor Nrf2 and its target HO-1 expression in A2780s and SKOV3 cells (Figure 3F). Because Nrf2/HO-1 signaling is a key antioxidant pathway that acts as a primary cellular defense system in response to oxidative stress [35, 36], we speculated that PUMA-stimulated Nrf2/HO-1 signal provides some protection against ROS-mediated oxidative stress in ovarian cancer cells.

### Generation of ROS leads to DNA damage response in ovarian cancer cells

**Figure 4:**
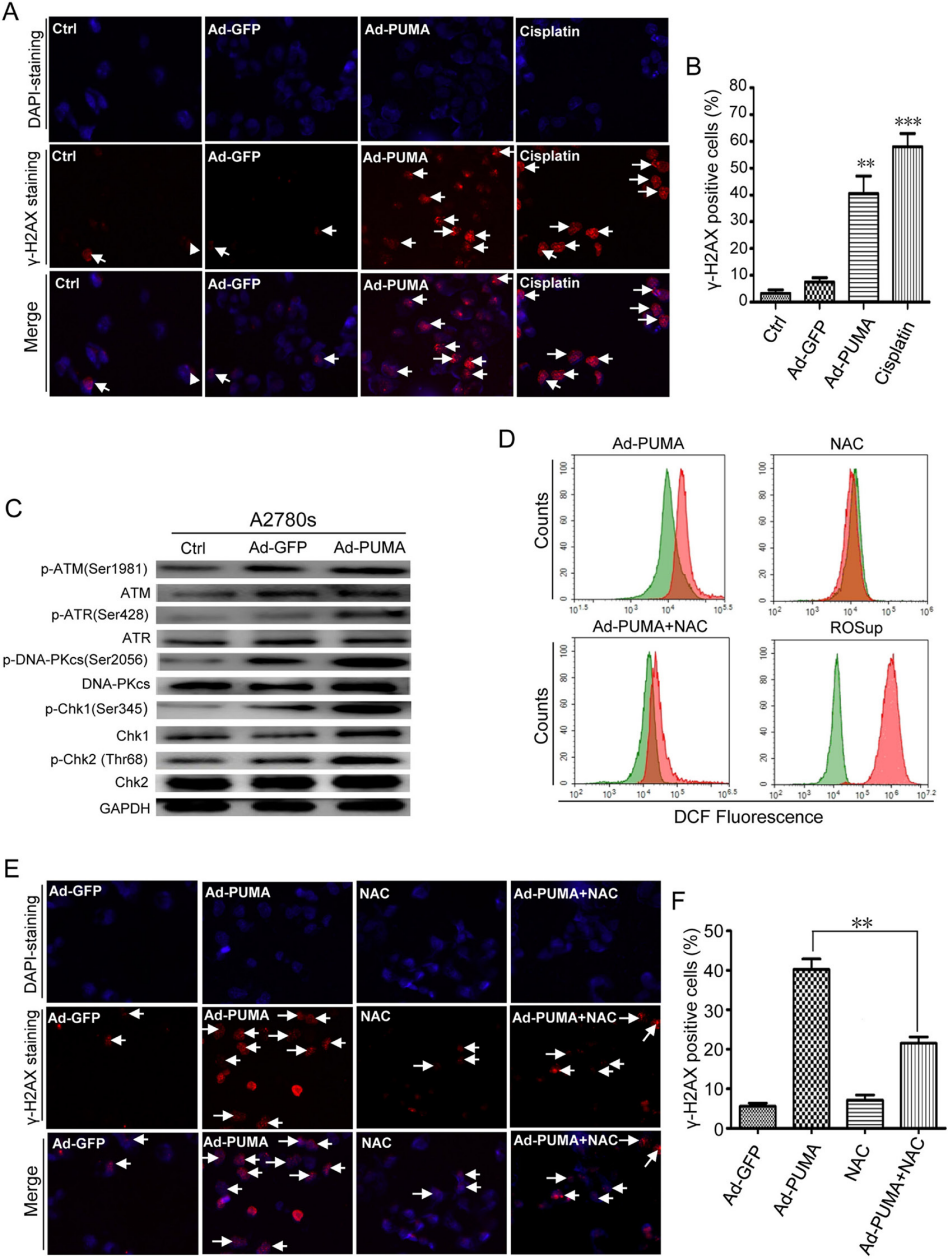
Detection of ROS-triggered DNA damage and its abrogation by NAC (**A**) Formation of γ-H2AX damage foci after treatment with PUMA or cisplatin (to provide a positive control). The representative pictures of PUMA/Cisplatin-induced γ-H2AX foci were analyzed in the indicated A2780s cells infected with PUMA adenovirus for 24 h or treated with 10 μM cisplatin for 12 h. Arrows, typical γ-H2AX foci. (**B**) Quantification of PUMA/Cisplatin-induced γ-H2AX foci. The percentage of γ-H2AX foci-positive cells (at least 300 total cells) was counted. Bars, mean; error bars, S.D. (*n* = 3; **p < 0.01; ***p < 0.001). (**C**) A2780s cells were infected with PUMA adenovirus for 36 h, and then the levels of total and phosphorylated ATM, ATR and DNA-PKcs, Chk1 and Chk2 in both cells were analyzed by western blotting. GAPDH was used as a loading control. (**D**) A2780s cells were infected with control Ad-GFP or PUMA adenovirus for 36 h in the presence or absence of 1 μM NAC, or treated with ROSup for 8 h (to provide a positive control). Then the treated cells were used for measuring ROS level by DCF fluorescence with flow cytometry. The DCF fluorescence shift of ROS in different groups (red) is compared with that of GFP control adenovirus (green). (**E**) A2780s cells were treated as described in D, and then γ-H2AX foci formation was detected by fluorescence microscopy. (**F**) Quantification of PUMA and/or NAC-induced *γ*-H2AX foci in E. The percentage of γ-H2AX foci-positive cells (at least 300 total cells) was counted. Bars, mean; error bars, S.D. (*n* = 3; **p < 0.01).

Since ROS can cause DNA damage response (DDR) [37, 38], we then tested if PUMA caused DDR in A2780s and SKOV3 cells. As expected, PUMA produced some typical *γ-H2AX foci*. In contrast, control adenovirus only produced few *γ-H2AX foci* (Figure 4A and 4B), and had little effect on the level of *γ-*H2AX (Supplementary Figure 3A and 3B). These results suggested that PUMA indeed induces an apparent DNA damage response.

ATM, ATR, DNA-PKcs, Chk1 and Chk2 kinases were activated when PUMA was overexpressed in A2780s (Figure 4C) and SKOV3 cells (Supplementary Figure 3C), suggesting that these checkpoint-related kinases were involved in the DDR. We further showed that, in A2780s cells, NAC significantly inhibited PUMA-induced ROS generation (Figure 4D). Notably, NAC also prevented the formation of *γ-*H2AX foci (Figure 4E and 4F). Additionally, Z-VAD-FMK abrogated PARP cleavage but did not prevent formation of *γ-H2AX* (Supplementary Figure 3D). Our data suggested that PUMA-induced DDR was not induced upon DNA fragmentation but resulted from ROS.

### ROS contributes to PUMA-induced proliferation inhibition

**Figure 5:**
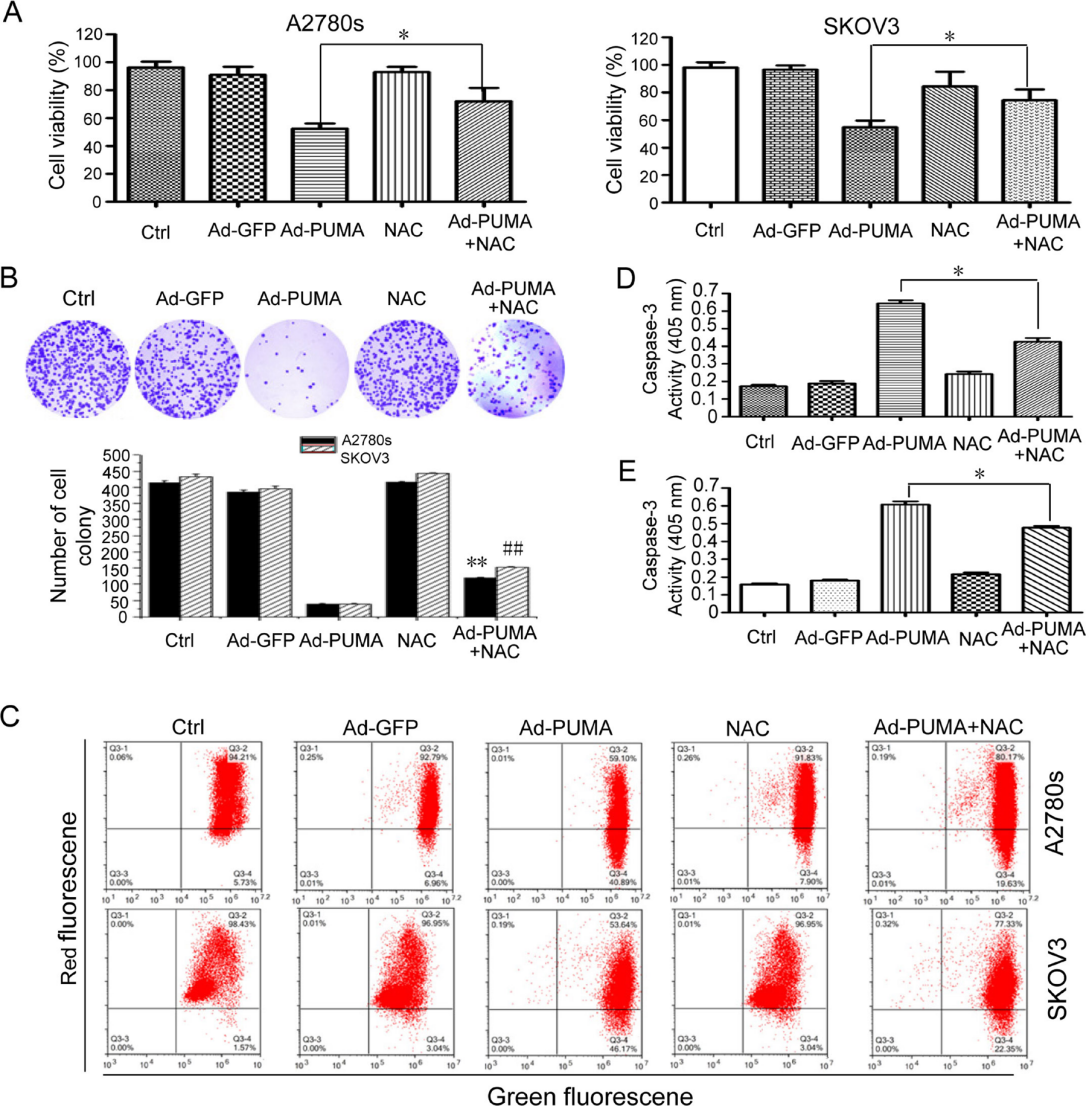
Role of PUMA-induced ROS in the proliferation inhibition of ovarian cancer cells (**A**) A2780s and SKOV3 cells were infected with PUMA adenovirus for 48 h in the presence or absence of 1 μM NAC. Then MTT was done to analyze the viability of the cells. PUMA significantly reduced the viability of A2780s and SKOV3 cells, compared with the control group (*P < 0.05). The percentage of survival was calculated. Results are shown as means *±* S.D. of three wells and triplicate experiments. In each experiment, the medium-only treatment (untreated) indicates 100% cell viability. (**B**) A2780s and SKOV3 cells were treated as described in A. Then colony formation assays were used to evaluate the cell viability. Representative pictures of A2780s cell colony formation were shown (top panel). Abrogation of ROS by NAC partially abolished the inhibition of PUMA on the proliferation of A2780s and SKOV3 cells (bottom panel). Bars, mean; error bars, S.D. (*n* = 3, **p < 0.01; ^##^*p* < 0.01). (**C**) A2780s and SKOV3 cells were treated as described in A. The treated cells were then used to measure the membrane potential by JC-1 dye retention using flow cytometry. (**D**) A2780s cells were treated as described in A. The treated cells were then lysed and caspase 3 activity was measured using an assay kit. Bars, mean; error bars, S.D. (*n* = 3, *p < 0.05). (**E**) SKOV3 cells were treated as described in A. The treated cells were then lysed and caspase 3 activity was measured using an assay kit. Bars, mean; error bars, S.D. (*n* = 3, *p < 0.05).

A2780s and SKOV3 cells were treated with PUMA adenovirus combined with NAC for an indicated time, then used for analyzing the rate of cell proliferation. As shown in Figure 5, PUMA significantly suppressed cell proliferation *via* reduction of viability, disruption of mitochondrial membrane potential and induction of apoptosis, as evidenced by MTT (Figure 5A), colony formation assays (Figure 5B), JC-1 staining (Figure 5C) and caspase-3 activity assay (Figure 5D and 5E). Whereas NAC partially abolished these suppressive effects of PUMA on the cell proliferation (Figure 5). Collectively, these data suggested that PUMA-induced ROS has an inhibitory role in cell proliferation.

### ROS-mediated DNA damage response is correlated with PUMA-induced apoptosis

**Figure 6:**
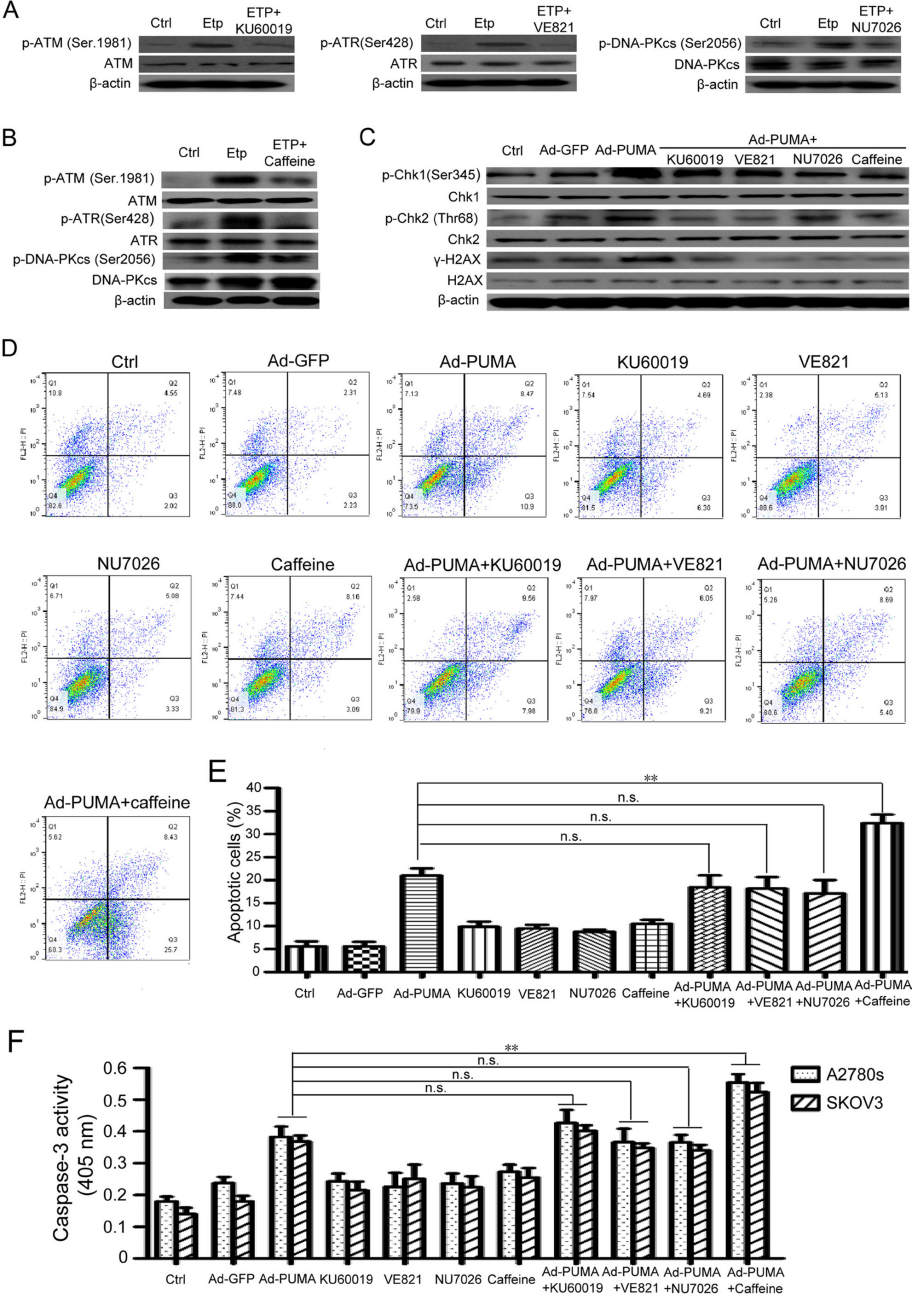
ROS-triggered DNA damage response is correlated with PUMA-induced apoptosis (**A**) Inhibition effects of ATM/ATR/DNA-PK inhibitors were confirmed by western blotting. A2780s cells were untreated or were treated with 10 μM etoposide (to provide a positive control) in the presence or absence of ATM-specific inhibitor KU60019 (5 μM), ATR-specific inhibitor VE821 (1 μM) and DNA-PK-specific inhibitor NU7026 (10 μM) for 24 h, and then the levels of total and phosphorylated ATM, ATR, and DNA-PKcs were detected by western blotting. β-actin was used as a loading control. (**B**) Inhibition effects of caffeine were confirmed by western blotting. A2780s cells were untreated or were treated with 10 μM etoposide (to provide a positive control) in the presence or absence of PIKKs inhibitor caffeine (2 mM) for 24 h, and then the levels of total and phosphorylated ATM, ATR, and DNA-PKcs were detected by western blotting. β-actin was used as a loading control. (**C**) Effect of ATM/ATR/DNA-PK inhibitors on the phosphorylation of Chk1, Chk2 and H2AX. A2780s cells were untreated or infected with Ad-PUMA for 24 h in the presence or absence of KU60019 (5 μM), VE821 (1 μM), NU7026 (10 μM) and caffeine (2 mM) for an additional 12 h, and then the expressions of total and phosphorylated Chk1 and Chk2 and H2AX were detected by western blotting. *β-*actin was used as a loading control. (**D**) A2780s cells were treated as described in B. Then the treated cells were used for analysis of apoptosis by flow cytometry. (**E**) The percentage of apoptotic cells was quantified. Bars, mean; error bars, S.D. (*n* = 3; **p < 0.01; N.S., not significant). (**F**) A2780s cells were treated as described in B. The treated cells were then lysed and caspase 3 activity was measured using an assay kit. Bars, mean; error bars, S.D. (*n* = 3; **p < 0.01; n.s., not significant).

Since PUMA-induced ROS generation can cause DNA damage response in ovarian cancer cells, we further investigated whether the DNA damage has an effect on PUMA-induced apoptosis. Cells were treated with PUMA adenovirus combined with the major DDR kinases inhibitor caffeine or single ATM-specific inhibitor (Ku60019), ATR-specific inhibitor (VE821) and DNA-PK-specific inhibitor (NU7026) for an indicated time. Then we analyzed the DNA-damaging effects on the apoptosis of A2780s and SKOV3 cells. KU60019, VE821 and NU7026 partially inhibited the activities of their corresponding kinase (Figure 6A). Whereas caffeine significantly inhibited the activities of all the three kinases (Figure 6B). We further tested the effects of KU60019, VE821, NU7026 and caffeine on the phosphorylation of *γ*-H2AX and Chk1 and Chk2, the two downstream checkpoint kinases of ATM, ATR and DNA-PK. All these inhibitors partially abrogated PUMA-induced Chk1 and Chk2 phosphorylations, and downregulated *γ*-H2AX (Figure 6C), suggesting that the DNA damage-related signaling were partially impaired. Surprisingly, three single DNA-damaging kinases inhibitors KU60019, VE821 and NU7026 have little effect, however, caffeine partially increased the apoptosis, as indicated by flow cytometry analysis (Figure 6D and 6E) and caspase 3 activity assay (Figure 6F). These data showed that ROS-mediated DNA damage is correlated with PUMA-induced apoptosis.

### Activation of JNK triggered by ROS mediates DNA damage response and contributes to PUMA-induced apoptosis

**Figure 7:**
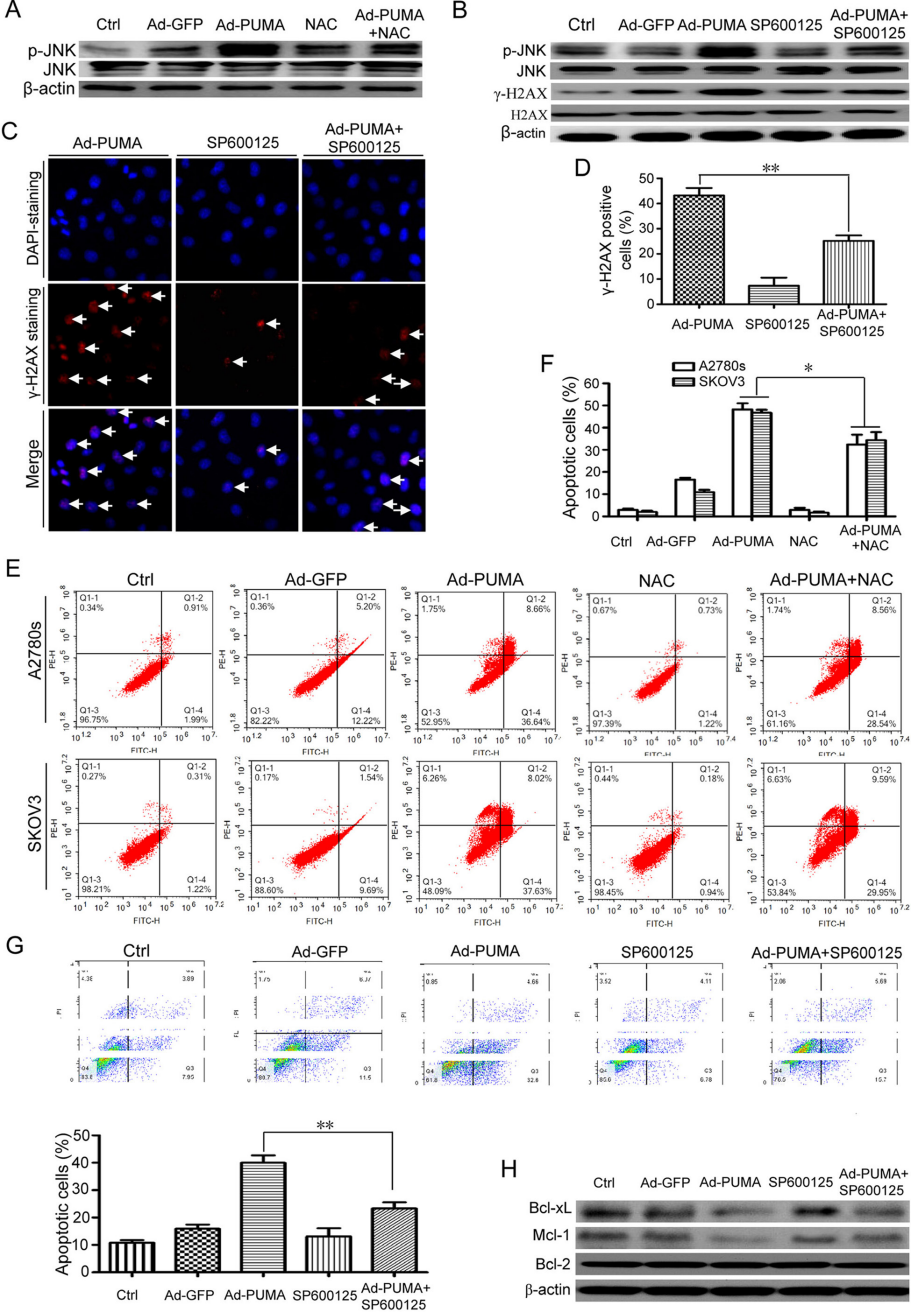
ROS-triggered JNK activation mediates DNA damage response and contributes to PUMA-induced apoptosis (**A**) A2780s cells were untreated or infected with Ad-PUMA for 36 h in the presence or absence of 1 μM NAC, and then the expressions of total and phosphorylated JNK were detected by western blotting. β*-*actin was used as a loading control. (**B**) A2780s cells were infected with PUMA adenovirus for 36 h in the presence or absence of 10 μM SP600125, and then the expressions of phosphorylated JNK and H2AX (γ-H2AX) were detected by western blotting. β-actin was used as a loading control. (**C**) A2780s cells were infected with PUMA adenovirus for 36 h in the presence or absence of 10 μM SP600125, and then γ-H2AX foci formation was determined by immunofluorescence staining. (**D**) Quantification of PUMA and/or SP600125-induced γ-H2AX foci in C. The percentage of γ-H2AX foci-positive cells (at least 300 total cells) was counted. Bars, mean; error bars, S.D. (*n* = 3; **p < 0.01). (**E**) A2780s and SKOV3 cells were infected with PUMA adenovirus for 36 h in the presence or absence of 1 μM NAC, and then the treated cells were used for analysis of apoptosis by flow cytometry. (**F**) The percentage of apoptotic cells was quantified. Bars, mean; error bars, S.D. (*n* = 3; *p < 0.05). (**G**) A2780s cells were infected with PUMA adenovirus for 36 h in the presence or absence of 10 μM SP600125, and then the treated cells were used for analysis of apoptosis by flow cytometry (top). The percentage of apoptotic cells was quantified (bottom). Bars, mean; error bars, S.D. (*n* = 3; **p < 0.01). (**H**) A2780s cells were treated as described in B, and then the expressions of MCL-1, BCL-2 and BCL-XL were detected by western blotting. *β-*actin was used as a loading control.

Previous studies have shown that MAP kinase JNK could be activated upon inhibition of Thioredoxin reductase (TrxR), a ROS-generating inhibitor [38, 39], implying that ROS could activate JNK [38]. Similarly, PUMA adenovirus caused JNK phosphorylation in A2780s cells (Figure 7A). Notably, the phosphorylation was triggered by ROS, as NAC partially impaired JNK activation (Figure 7A).

Because JNK mediates the formation of *γ*-H2AX [40], SP600125, a JNK inhibitor, was used to evaluate the action of JNK in the DNA damage response. SP600125 significantly inhibited p-JNK and *γ*-H2AX (Figure 7B), and markedly reduced formation of *γ*-H2AX foci (Figure 7C and 7D), suggesting that JNK is one of the key mediators for the DNA damage response. Additionally, we found that ROS-triggered JNK activation also plays a positive role in PUMA-induced apoptosis, as NAC (Figure 7E and 7F) and SP600125 (Figure 7G) partly abrogated PUMA-induced apoptosis. We further explored the action mechanism of JNK. Strikingly, PUMA-induced downregulation of Bcl-xL and Mcl-1 (Figure 7H), which was similar to our previous observations in ovarian cancer cells [2], were partially rescued by JNK inhibitor SP600125 (Figure 7H).

## DISCUSSION

PUMA is a downstream molecule of p53 and p73, and has strong killing activity against many cancer cells via apoptosis [2, 4, 15, 16, 22–25]. Our previous study showed that PUMA could chemosensitize ovarian cancer cells [2], However, the action mechanism remains elusive. A previous report has shown that PUMA overexpression induces ROS generation in colorectal cancer cells [26]. Nevertheless, the role of ROS remains to be elucidated. This work was to investigate if ROS was also generated in ovarian cancer cells overexpressing PUMA, and to elucidate the action of ROS.

Neither PUMA up-regulation nor high levels of PUMA could induce apoptosis if it was kept in the cytosol [29]. However, when translocation to the mitochondria, PUMA immediately induced apoptosis [29]. Similarly, exogenous PUMA was found to be accumulated in the cytosol and mainly in the mitochondria in PUMA adenovirus-infected SKOV3 cells (Figure 1B). Thus, we speculated that accumulation in the cytosol and translocation to the mitochondria of exogenous PUMA might be a prerequisite for triggering apoptosis.

A previous report has shown that PUMA overexpression induces ROS generation in colorectal cancer cells [26]. Similarly, PUMA also induces ROS generation independently of p53 in ovarian cancer cells (Figure 2A and 2B). Generation of ROS is either mitochondrial or enzyme dependent and affects several vital cellular functions, including differentiation and intracellular signaling [33, 41]. In this work, we found that PUMA-induced ROS mainly originated in mitochondria, and that ROS is generated in a ROS generating enzyme-independent manner, as evidenced by MitoSOX fluorescence staining (Figure 2C) and western blot for the levels of NOX1, NOX4, Duox1 and SOD1 (Figure 2F). Furthermore, PUMA induces ROS generation through functional BAX, as indicated by block of ROS generation by a BAX-inhibiting peptide (Figure 2D and 2E). Because ROS generation may be caused by caspase activation [33], we treated ovarian cancer cells with Ad-PUMA plus Z-VAD-FMK, and found that ROS did not significantly decreased (Figure 3A and 3B), thus ruling out the possibility that PUMA-induced ROS generation is a consequence of caspase activation.

Nrf2/HO-1 signaling pathway is involved in regulating mitochondrial ROS production [42], implying that PUMA-induced ROS might be associated with alteration of this pathway. In this work, we observed that PUMA markedly up-regulated the levels of Nrf2 and HO-1 (Figure 3F). Nrf2/HO-1 axis is vital for protecting cell against some damaging stresses [34–36]. Nrf2 is regulated by Keap1 [42]. Furthermore, Nrf2 showed its antioxidant properties when stimulating the transcription of HO-1 [43]. Based on these previous observations, we speculated that PUMA-activated Nrf2/HO-1 signal is an antioxidant response to ROS-mediated oxidative stress.

Previous studies have shown that ROS causes DNA damage response [37, 38]. Similarly, we found that PUMA induced an apparent ROS-mediated DNA damage response, as evidenced by *γ-H2AX* staining (Figure 4A and 4B), an increased phosphorylation of H2AX (Supplementary Figure 3A and 3B) and inhibition of both ROS production (Figure 4D) and *γ-H2AX* foci formation by NAC (Figure 4E and 4F). Furthermore, the key checkpoint kinases in the DNA damage pathway including ATM, ATR, DNA-PKcs, Chk1 and Chk2 were involved in the DNA damage response (Figure 4C and Supplementary Figure 3C). In addition, we ruled out the possibility that the DNA damage response was associated with DNA fragmentation during apoptosis, as Z-VAD-FMK prevent PARP cleavage (Supplementary Figure 3D), but did not prevent phosphorylation of *γ-H2AX* (Supplementary Figure 3D). Next, we focus on the action of ROS in cell proliferation inhibition by PUMA. We found that PUMA significantly inhibit cell proliferation *via* reduction of viability (Figure 5A and 5B), disruption of mitochondrial membrane potential (Figure 5C) and induction of apoptosis (Figure 5D–5E). However, NAC partially abolished the inhibition of PUMA on the cell proliferation (Figure 5), suggesting that ROS contributes to the inhibition of PUMA on ovarian cancer cells. Then we further investigated the role of ROS-mediated DNA-damaging effects on the apoptosis. We observed that single chemical reagent KU60019 (ATM inhibitor), VE821 (ATR inhibitor) or NU7026 (DNA-PK inhibitor) have a little effect on PUMA-induced apoptosis, however, PUMA-induced apoptosis was further stimulated by caffeine (Figure 6), implying that the DNA-damaging effects of a single kinase inhibitor could be complemented by that of other kinase inhibitors, and that ROS-mediated DNA damage might be correlated with PUMA-induced apoptosis.

Previous studies have shown that ROS could activate JNK [38, 39]. Similarly, PUMA also induced JNK phosphorylation (p-JNK) (Figure 7A). Furthermore, p-JNK was enhanced by ROS, because NAC partly impaired JNK activation (Figure 7B). These data showed that ROS contributes to JNK activation. As JNK mediated the formation of *γ*-H2AX foci and pan-nuclear apoptotic staining of *γ*-H2AX, suggesting an important role of JNK in DNA damage response and apoptosis [40]. Similarly, JNK inhibitor SP600125 markedly reduced *γ*-H2AX foci (Figure 7C and 7D), suggesting a positive role of JNK in PUMA induced- DNA damage response. Furthermore, SP600125 and NAC partially abrogated PUMA-induced apoptosis (Figure 7E–7G). Furthermore, PUMA-induced decrease of Bcl-xL and Mcl-1 in ovarian cancer cells were partially rescued by SP600125 (Figure 7H). Taken together, our data showed that JNK has a dual function in both DNA damage response and apoptosis, finally contributing to apoptosis.

In summary, our data demonstrate that exogenous PUMA induced mitochondria ROS generation through functional BAX in a p53- and ROS generating enzyme-independent manner, which in turn results in DNA damage response and activation of JNK, finally contributing to apoptosis. Our data also show that PUMA induces the activation of Nrf2/HO-1 pathway, which may be an antioxidant response to ROS-mediated oxidative stress. In addition, our data suggest that JNK has dual functions in DNA damage response and apoptosis, and has an additional role in enhancing PUMA-induced apoptosis. To our knowledge, we provided new insight into the pro-aopptotic mechanism for PUMA.

## MATERIALS AND METHODS

### Recombinant adenoviruses and infection

PUMA adenoviruses (Ad-PUMA) were constructed using the Ad-Easy system as previously described [18]. Control adenovirus (Ad-GFP) expressed a foreign GFP gene. High-titer viruses were produced in 293A cells and purified by cesium chloride gradient centrifugation as described previously [44]. Ovarian cancer cells were infected with Ad-GFP or Ad-PUMA with a range of concentrations from 10 to 20 multiplicity of infection (MOI) (or plaque-forming units per cell, which was estimated to be 20–400 virus particles per cell) for indicated times.

### Reagents and chemicals

MTT, dimethyl sulfoxide (DMSO), PI, DAPI and DCFH-DA were purchased from Sigma (St Louis, MO). RIPA buffer and JC-1 staining kit were purchased from Beyotime Biotechnology, China. The following antibodies were used: anti-PUMA, anti-p53, anti-Chk1, anti-Chk2, anti-p-Chk1(Ser345), anti-p-Chk2 (Thr68), anti-ATM, anti-p-ATM (Ser1981), anti-PARP, anti-GAPDH and anti-β-actin (Santa Cruz Technology, Santa Cruz, CA, USA); Anti-NOX1, anti-NOX4, anti-DUOX1 and anti-SOD1, anti-Keap1, anti-Nrf2, anti-HO-1, anti-DNA-PKcs, anti-p-DNA-PKcs (Thr 2609), anti-Caspase-3 and anti-Caspase-9 (Cell Signaling Technology, Danvers, MA, USA); Anti-*γ* H2AX (Ser139), anti-H2AX and anti-JNK, anti-p-JNK, anti-BCL-XL, anti-MCL-1 and anti-BCL-2 (Abcam). Rhodamine (TRITC) AffiniPure Goat anti-Rabbit IgG was from Santa Cruz Biotechnology; KU-60019, VE-821 and NU7026 were obtained from Selleck (USA). SP600125 were obtained from Calbiochem (La Jolla, CA, USA). Z-VAD-fmk was purchased from R&D Systems (Minneapolis, MN). These inhibitors were dissolved in DMSO and diluted to appropriate concentrations with cell culture media. Mitotracker and MitoSOX red mitochondrial superoxide indicator was purchased from Molecular Probe (USA).

### Cell culture and treatments

Human A2780s, A2780cp, OVCAR-3 and SKOV3 ovarian cancer cell lines and HEK 293A cells were obtained from the ATCC (Manassas, VA). Cells were cultured in RPMI 1640 or DMEM medium (GIBCO). All culture medium were supplemented with 10% FBS, penicillin (100 units/ml) and streptomycin (100 μg/ml), respectively, at 37°C in a humidified atmosphere containing 5% CO_2_. For single adenovirus infection, cells were left untreated or infected with adenoviruses including Ad-GFP (control) and Ad-PUMA. For combination treatments, cells were infected with adenoviruses for 24 to 48 hours before treatment with other inhibitor. After treatments, cells were harvested and used for the following experiments including MTT assay, Colony formation assays, flow cytometric analysis, measurement of ROS and mitochondrial membrane potential (ΔΨ), immuno- fluorescence microscopy and western blotting analysis.

### Cell viability and apoptosis assays

Cell viability was assessed by MTT assay [9, 45]. Briefly, cells were plated in 96-well plates at 5 × 10^3^ cells /well and incubated at 37°C in 5% CO_2_, 95% humidity air for 24 h, and then cells were treated as described above. MTT was added to the medium (0.5 mg/ml) and incubated at 37°C for 4 hours. The resulting insoluble formazan was dissolved with DMSO and measured at 570 nm using a spectrophotometer. Data represent the average of three wells, and the experiment was repeated three times. Apoptosis was assessed by flow cytometry analysis, by detecting the caspase-3 activity and cleavages of caspase-3, -9 and PARP.

### Flow cytometry analysis

Cells were collected and pelleted at 1,500 rpm for 3 min and suspended in 1 ml of hypotonic fluorochrome solution containing 50 μg/ml PI and Annexin in 0.1% sodium citrate plus 0.1% Triton X-100, then were analyzed by the use of a flow cytometer (ESP Elite, Coulter, Miami, FL).

### Colony formation assays

Colony formation assays were conducted as described previously [46]. Briefly, A2780s and SKOV3 cells infected with adenoviruses for 48 h were plated in triplicate at 500 cells /well in 6-well plates and cultured for 10 days. Then the treated cells were washed twice in PBS, fixed in cold methanol, and stained with 2% crystal violet. After incubation at room temperature for 20 min, the 6-well plates were washed twice in double-distilled H_2_O and dried, and colonies containing more than 50 cells were counted. All of the experiments were repeated three times, and the average values were reported.

### Measurement of caspase 3 activity

Caspase 3 activity was analyzed using a colorimetric test system as described previously [31]. Briefly, cells were infected with Ad-PUMA adenoviruses for 24–48 h and 1 *×* 10^6^ cells were lysed with lysis buffer on ice. The lysates were centrifuged at 14,000 g at 4°C for 15 min. Cytosolic protein was mixed with 10 μl caspase 3-specific substrate acetyl-Asp-Glu-Val-Asp p-nitroanilide (2 mM) for 2 h at 37°C. The value of absorbance of sample was monitored at 405 nm using a Spectramax M5 Microtiter Plate Luminometer.

### Measurement of ROS

DCFH-DA was used to measure ROS formation. After infection with adenoviruses for 24–48 h, cells were incubated in 10 μM DCFH-DA containing medium at 37°C for 20 min. Cells were then washed with PBS three times to remove DCFH-DA, which had not entered into the cells. The fluorescence was visualized immediately at wavelength of 485 nm for excitation and 530 nm for emission by an inverted fluorescence microscope. Total green fluorescence intensities of each well were quantified using image-analysis software (Cell Quest Program). The level of ROS was also measured by DCF fluorescence with flow cytometry.

### Measurement of mitochondrial membrane potential (ΔΨ)

Mitochondrial membrane potential was measured by 5, 5′, 6, 6′-tetrachloro-1, 1′, 3, 3′- tetraethylbenzimidazolocarbocyanine iodide (JC-1) dye retention using flow cytometry. The cells infected with adenoviruses for 48 h were incubated with JC-1 at 37°C for 30 min, then collected and washed twice with cold PBS. The monomeric form emits light at 527 nm after excitation at 490 nm. Thus, the color of the dye changes from orange to green, depending on the mitochondrial membrane potential, and could be analyzed by flow cytometry with orange emission.

### Western blot

Protein samples were mixed with loading buffer (100 mM Tris-HCl, pH 6.8, 2% SDS, 100 mM dithiothreitol, 0.01% bromphenol blue, and 10% glycerol), heated at 100°C for 5 min, and loaded onto a 12% SDS-PAGE in electrophoresis buffer containing 25 mM Tris-HCl, pH 8.3, 250 mM glycine, and 0.1% SDS. Protein was then transferred to PVDF using the Trans-Blot semidry system (Bio-Rad). The membranes were immunoblotted with primary antibodies as described under “Reagents and chemicals.”

### Indirect immunofluorescence microscopy

A2780s and SKOV3 cells were untreated or infected with Ad-GFP (control) and Ad-PUMA adenoviruses, and incubated in a 5% CO_2_ incubator at 37°C for 24 h. After fixation with methanol at −20°C, the cells were immunostained with monoclonal anti-γ-H2AX (Ser-139) antibodies and subsequently with rhodamine (TRITC) affinipure goat anti-rabbit IgG and counterstained with DAPI (Invitrogen). The fluorescence images were visualized with a Zeiss fluorescence microscope.

### Statistical methods

The statistical analysis was performed with SPSS software (version 17.0 for Windows). Results are presented as mean ± S.D. Analysis of variance and the Tukey-Kramer multiple-comparison test were used in comparisons. *P<0.05* was considered to be statistically significant.

## SUPPLEMENTARY MATERIALS FIGURES


