# Supplementary material for: The role of ROS and subsequent DNA-damage response in PUMA-induced apoptosis of ovarian cancer cells
Source: Oncotarget. 2017 Feb 22;8(14):23492–506. doi: 10.18632/oncotarget.15626 (PMC5410321; doi:10.18632/oncotarget.15626)
Supplement: Supplementary file 1 [file oncotarget-08-23492-s001.pdf]

The role of ROS and subsequent DNA-damage response in PUMA-induced apoptosis of ovarian cancer cells

Supplementary Materials

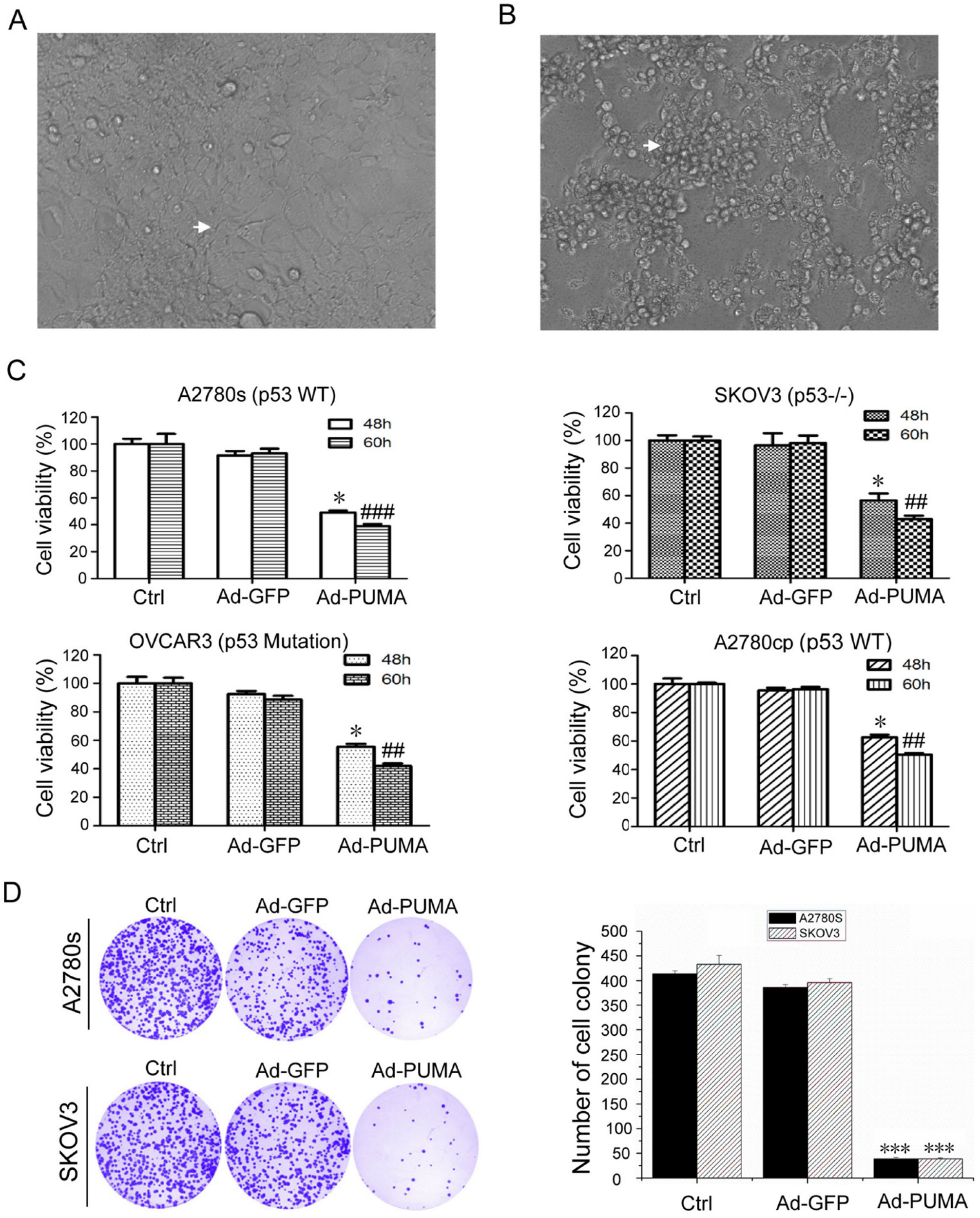

Supplementary Figure 1: PUMA reduces viability of ovarian cancer cells. (**A**) The morphological change of HEK293A cells before infection with PUMA adenovirus for 36h. Arrows, typical cell morphology. (**B**) The morphological change of HEK293A cells after infection with PUMA adenovirus for 36 h. Arrows, typical cell morphology. (**C**) MTT was done to analyze the viability of ovarian cancer cells. PUMA overexpression significantly reduced the viability of A2780s, SKOV3, OVCAR3 and A2780cp cells, compared with the control group (**P* < 0.05; ##*P* < 0.01 and ###*P* < 0.001). The percentage of survival was calculated. Results are shown as means ± S.D. of three wells and triplicate experiments. In each experiment, the medium-only treatment (untreated) indicates 100% cell viability. (**D**) Colony formation assays were further used to evaluate the viability of A2780s and SKOV3 cells. PUMA overexpression resulted in significant inhibition of clone formation compared with the control and Ad-GFP groups. Bars, mean; error bars, S.D. (*n* = 3, ****p* < 0.001).

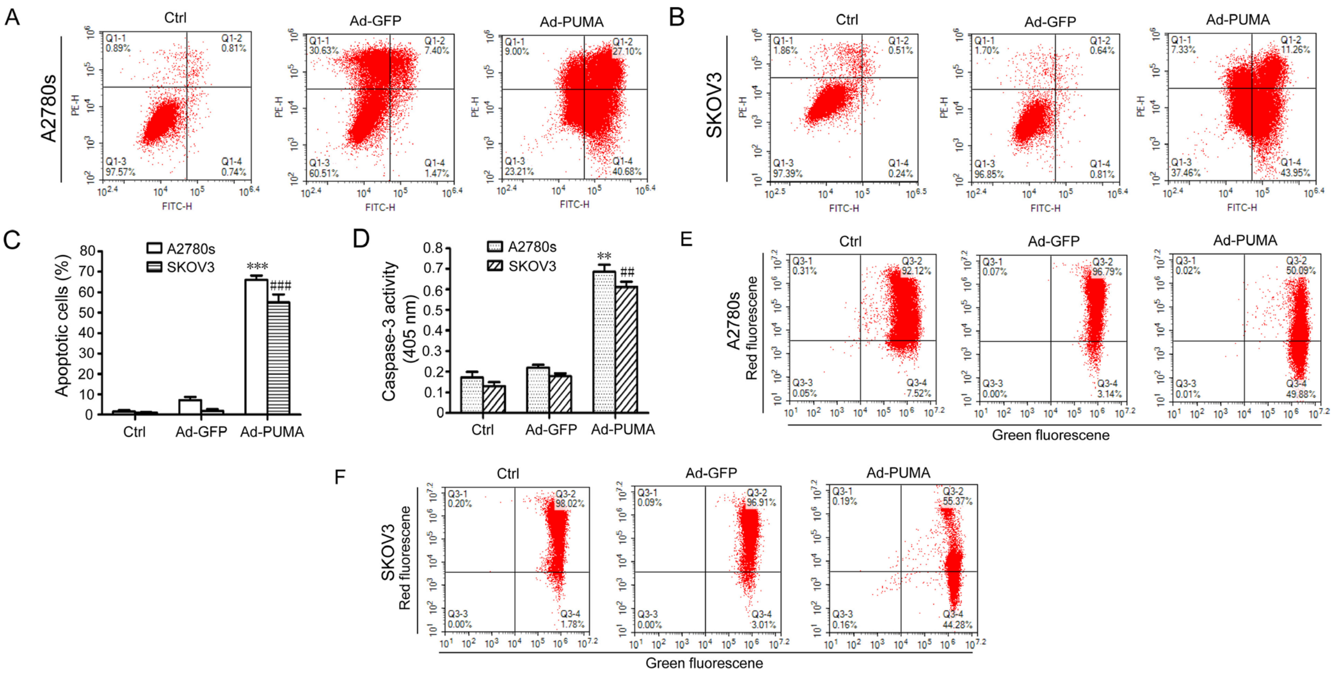

Supplementary Figure 2: PUMA induces apoptosis via mitochondrial apoptotic pathway. (**A**) A2780s cells were untreated or infected with Ad-GFP or Ad-PUMA for 60 h. The treated cells were used for analysis of apoptosis by flow cytometry. (**B**) SKOV3 cells were treated as described in A, then the treated cells were used for analysis of apoptosis by flow cytometry. (**C**) Apoptotic A2780s and SKOV3 cells were further counted. Bars, mean; error bars, S.D. ($n = 3$; $^{***}p < 0.001$; $^{###}p < 0.001$). (**D**) PUMA activated caspase-3 both in A2780s and SKOV3 cells. A2780s and SKOV3 cells were infected with Ad-GFP or Ad-PUMA for 60 h. The treated cells were then lysed and caspase 3 activity was measured using an assay kit ($n = 3$; $^{**}p < 0.01$; $^{##}p < 0.01$). (**E**) PUMA decreased the mitochondria membrane potential (*ΔΨ*) of A2780s cells. The cells were treated as described in C. The treated cells were used to measure the membrane potential by JC-1 dye retention using flow cytometry. (**F**) PUMA decreased the mitochondria membrane potential (*ΔΨ*) of SKOV3 cells. The cells were treated as described in C. The treated cells were used to measure the membrane potential by JC-1 dye retention using flow cytometry.

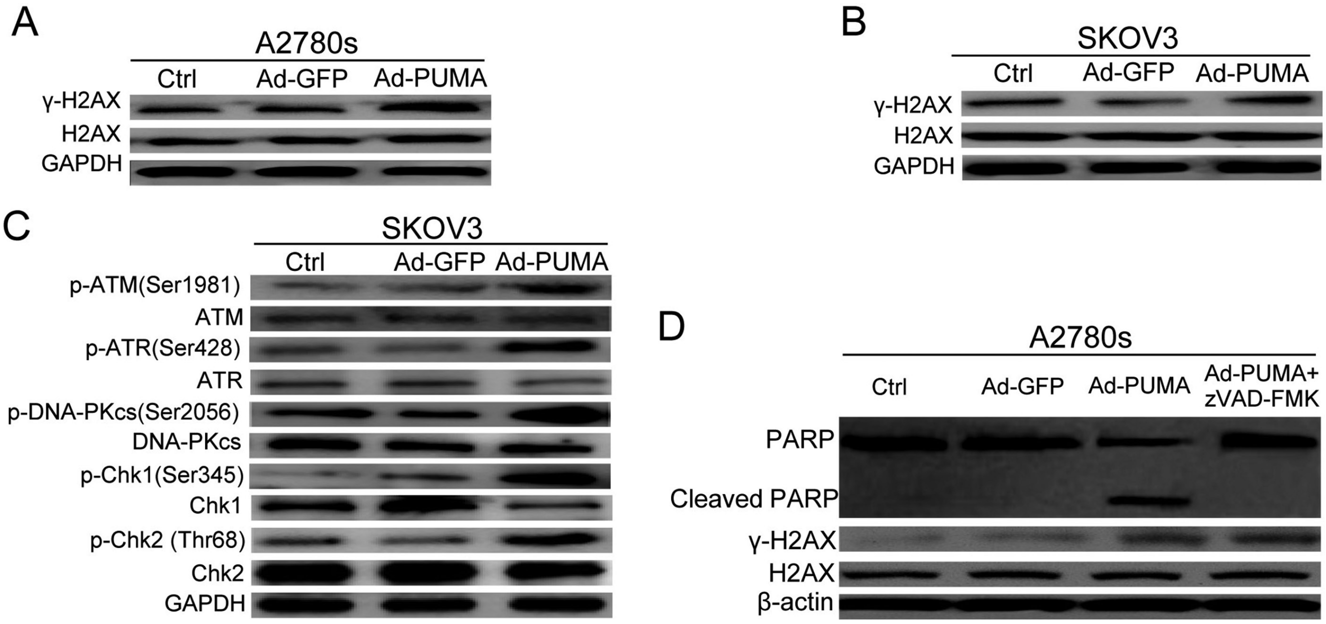

Supplementary Figure 3: Detection of ROS-triggered caspase-independent DNA damage. (**A**). A2780s cells were infected with PUMA adenovirus for 36 h, and then phosphorylated H2AX (γ-H2AX) and total H2AX in both cells were analyzed by western blotting. GAPDH was used as a loading control. (**B**) SKOV3 cells were infected with PUMA adenovirus for 36 h, and then phosphorylated H2AX (γ-H2AX) and total H2AX in both cells were analyzed by western blotting. GAPDH was used as a loading control. (**C**). SKOV3 cells were treated as described in B, and then the levels of total and phosphorylated ATM, ATR and DNA-PKcs, Chk1 and Chk2 in both cells were analyzed by western blotting. GAPDH was used as a loading control. (**D**) A2780s cells were infected with Ad-PUMA for 48 h in the presence or absence of 10 μM benzyloxycarbonyl-VAD-fluoromethyl ketone (z-VAD-FMK), and then cleavage of PARP, γ-H2AX and total H2AX were detected by western blotting. β-actin was used as a loading control.
